# Occlusal Plane Determination Using Custom Made Broadrick Occlusal Plane Analyser: A Case Control Study

**DOI:** 10.5402/2012/373870

**Published:** 2012-02-20

**Authors:** Supriya Manvi, Shaveta Miglani, C. L. Rajeswari, G. Srivatsa, Sarvesh Arora

**Affiliations:** Department of Prosthodontics and Crown and Bridge, K.L.E.V.K. Institute of Dental Sciences, Belgaum 590010, India

## Abstract

Proper occlusal plane is an essential consideration when multiple long span posterior restorations are designed. The determination of the occlusal plane can have a profound effect on the short and long term success of a restorative case. *Purpose of Study*. (1) To determine the appropriate occlusal curve for individual patients. (2) To compare the deviation of the clinical occlusal curve with the ideal ones. *Materials and Methods*. A total of 20 subjects were examined and study models were made of their maxillary and mandibular dentition. Inter-occlusal records were made and the casts were articulated in semiadjustable articulator. An ideal occlusal plane was created. The distance of the farthest cusp tip from the Broadrick curve was measured along the long axis of the tooth for each individual. Paired *t*-tests were used to compare the findings between subjects and controls. *Results*. A statistically significant difference *P* < 0.05 was found in the deviation from the Broadrick curve between patients who have lost posterior teeth and the control group who had a full dentition with no missing teeth. *Conclusion*. Proper utilization of the broadrick flag on a semi-adjustable articulator will allow for a correct determination of the occlusal plane.

## 1. Introduction

One of the most important decisions that a restorative dentist often has to make is the determination of a functional and esthetic occlusal plane. When the patient has missing or malaligned posterior teeth that need to be replaced, it is important that these teeth are positioned in the most ideal place for that patient. The curvature of the arch in three dimensions, including the curve of Spee and the curve of Wilson as well as the placement of the teeth, must be determined for treatment to be successful. An occlusal plane analyser has long been used to assist the operator in the development of an initial mandibular occlusal plane in diagnostic casts and later as an integral part of both the contours of the definitive restorations and the guidelines for the actual tooth preparations [1–3]. The curve of Spee may be pathologically altered in situations resulting from rotation, tipping, and extrusion of teeth. Restoration of the dentition to such an altered occlusal plane can introduce posterior protrusive interferences [4]. Such interferences have been shown to cause abnormal activity in mandibular elevator muscles, especially the masseter and temporalis muscle [5]. This can be avoided by reconstructing the curve of Spee to pass through the mandibular condyle, which has been demonstrated to allow posterior disocclusion on mandibular protrusion [6]. As the angle of condylar guidance is greater than curve of Spee, posterior disocclusion is achieved [7].

The Broadrick flag permits construction of the curve of Spee in harmony with anterior condylar guidance allowing total posterior tooth disclusion on mandibular protrusion. Its use assumes proper functional and esthetic positioning of the mandibular incisors [8].

## 2. Materials and Methods

 A total of 20 subjects were examined and study models of their maxillary and mandibular dentition were made. The study included 10 completely dentate patients (control group) with no missing teeth and 10 partially dentate patients with a few posterior teeth missing. Right and left sides of each patient were considered separately in the study. 

### 2.1. Selection Criteria

 Students and the patients from K.L.E.S.V.K. Institute of Dental sciences, Belgaum, were taken for the study. Adult patients of 18 years or above were selected. A written informed consent was obtained from all subjects following explanation of the purpose and methods to be used in the study. 

### 2.2. Exclusion Criteria

 Teeth that were used as R.P.D or F.P.D abutments were excluded from the study. 

### 2.3. Procedure

**Figure 1:**
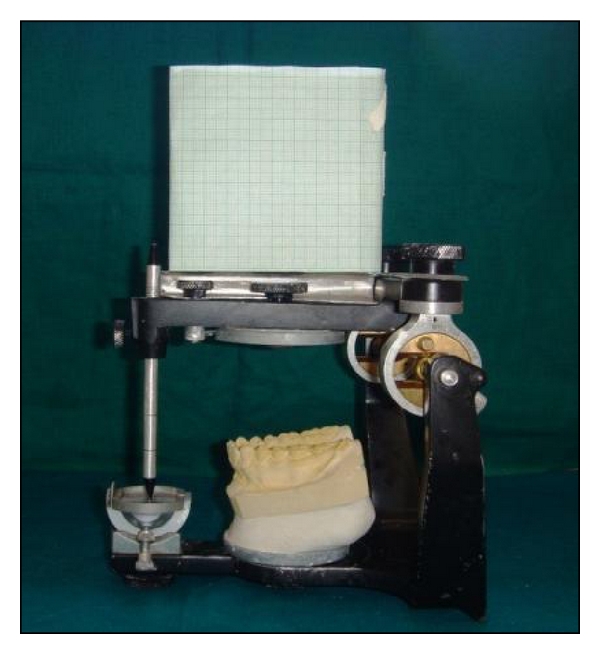
Custom made Broadrick occlusal plane analyzer attached on a semiadjustable articulator.

 Alginate impressions of the maxillary and mandibular dentition for all the 20 subjects were made. The face bow transfer was performed and maxillary cast was oriented on a semiadjustable articulator. Later, interocclusal records were made and the mandibular cast was articulated. The adaptation of the occlusal plane analyzer to the upper member of the semiadjustable articulator using the Broadrick flag method described by Lynch and McConnell [8] was done to create the ideal occlusal plane (Figure 1). 

**Figure 2:**
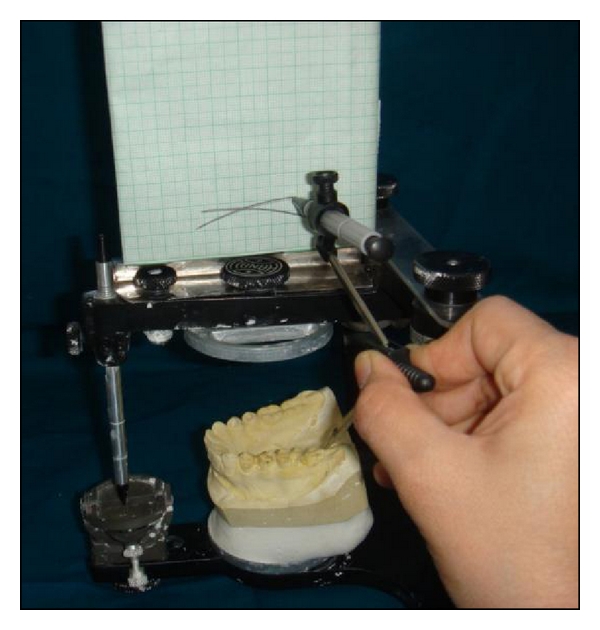
Anterior and posterior survey lines drawn together bisecting at central survey point.

**Figure 3:**
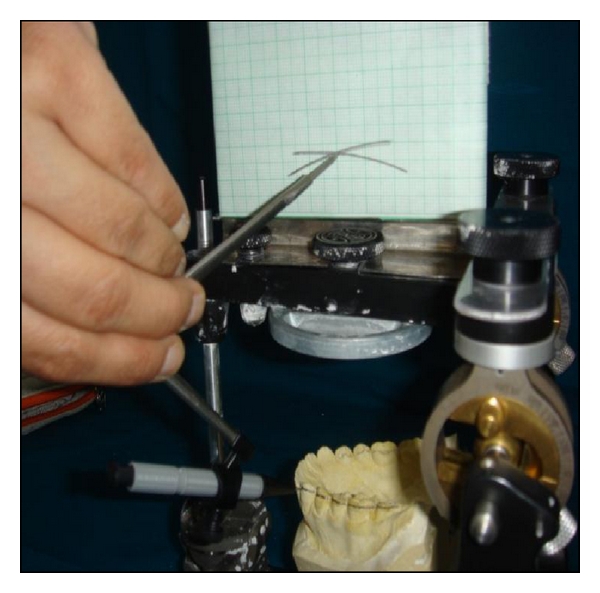
The central survey point was used to mark the curve on lower cast.

**Figure 4:**
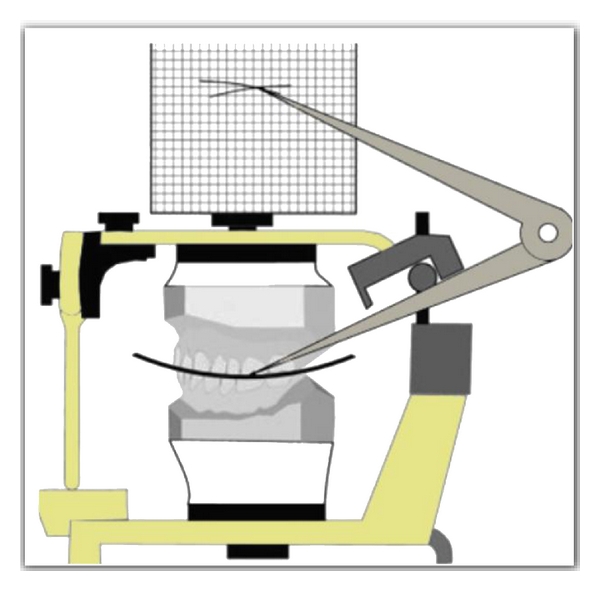
Graphic representation of the survey lines.

**Figure 5:**
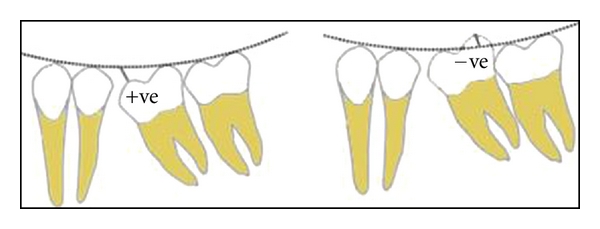
Where the deviation was outside the existing curve, a positive notation was given; if the deviation was inside the curve, a negative notation was given.

The anterior survey point, posterior survey point, and central survey point were located as follows. The anterior survey point was located on the distal slope of the lower canine tooth, from which a long arc with a four-inch radius was drawn on the flag with a compass, as anterior survey line. The posterior survey point was located on the distal slope of the distobuccal cusp of the lower second molar and a short arc was drawn on the flag to intersect the anterior survey line (Figure 2). Anterior and posterior survey lines bisect at central survey point. The point of the compass was placed at the centre of anterior and posterior survey lines (central survey point), and a 4-inch radius was drawn through the buccal surfaces of the mandibular teeth (Figures 3 and 4). Where the deviation was outside, the existing curve a positive notation was given; if the deviation was inside the curve, a negative notation was given (Figure 5). Paired *t*-tests were used to compare the findings between subjects and controls. Significance was assessed at the 0.05 level. 

## 3. Results 

 Deviation from the Broadrick curve was found to be marked in subjects who had missing posterior teeth, while fairly minimal in the control group (Tables 1 and 2). The results showed that there is a statistically significant difference (*P* < 0.05 level) in the deviation from the Broadrick curve between patients who have lost posterior teeth and the control group (Table 3). 

## 4. Discussion 

 The Broadrick Flag technique was designed to provide a guide to the location of the Curve of Spee, to facilitate the accurate determination of posterior occlusal plane. Acrylic template can be used to facilitate controlled conservative reduction. Template acts as a vital tool for transferring the designed blueprint from the diagnostic wax up on the articulator to the mouth. 

The Broadrick flag is a useful tool in a prosthodontic and restorative dentistry, as it identifies the most likely position of the center of the curve of Spee. However this position should not be regarded as fixed or immutable. Esthetics and function plays a considerable demand on the design of occlusal plane. Compromise can be achieved by altering the length of the radius of curve. In patients with a retrognathic mandible, a standard four-inch curve would result in a flat posterior curve, causing posterior protrusive interferences. Such “low” mandibular posteriors would also lead to extrusion of the opposing maxillary teeth. If the maxillary posterior teeth were to be restored to this low occlusal plane, the crown to root ratio would be less than ideal. Hence, 3.75-inch radius is more appropriate when a class II skeletal relationship exists. Conversely, a four-inch curve would create a steep posterior curve in a patient with a class III skeletal relationship, leading to further posterior interference. A five-inch radius would be more suitable in this situation [8]. 

The centre of the curve also may be varied to achieve the same effect. The centre should always lie along the long arc drawn from the anterior survey point, but it may be moved in an anterior or posterior direction from the intersection of this arc with that drawn from the posterior survey point. This alteration will not affect the position of the anterior survey point, an important fact when the position of the mandibular anterior teeth is esthetically and clinically suitable. Therefore with a little experience and training this aids the clinician for the definitive restorations as well as a guide for the actual tooth preparations [9, 10]. 

## 5. Summary 

 The determination of occlusal plane can have a profound effect on short- and long-term success of a restorative case. The less posterior interference that results from the reconstruction means fewer problems for patients with their teeth, muscles, and TMJ. The simple modification procedure enables the practitioners to use an occlusal plane analyzer as a diagnostic tool with a widely used semiadjustable articulator, which therefore had no such available accessory. This modification also serves as a practical teaching aid for students during treatment planning and subsequently for the planned rehabilitation of complete restorative situations that frequently exhibit loss of many occlusal landmarks. 

## 6. Conclusion 

 Proper utilization of the Broadrick flag on a semiadjustable articulator will allow easy determination of the occlusal plane for each case which will fulfill esthetic and functional occlusal requirements. With a little experience and training, dentists can make Broadrick flag an integral part of their practice. 

## Figures and Tables

**Table 1:** Deviations from the Broadrick curve in control group in three different locations on 2nd premolar, 1st molar, and 2nd molar.

S no.	2nd premolar (mm)	1st molar (mm)	2nd molar (mm)	Mean (mm)
1	0.5	0.5	0.5	0.5
2	0.5	1	1	0.8
3	0.5	1	0.5	0.6
4	1	1	0.5	0.8
5	1.5	0.5	0.5	0.6
6	1	1	0.5	0.8
7	1.5	1	1	1.2
8	1.5	1	0.5	1
9	1	1.5	1	1.2
10	1	1.5	1	1.2

Total				0.88

**Table 2:** Deviations from the Broadrick curve in subject group in three different locations on 2nd premolar, 1st molar, and 2nd molar.

S no.	2nd premolar (mm)	1st molar (mm)	2nd molar (mm)	Mean (mm)
1	1	1.5	0.5	1
2	1	1.5	0.5	1
3	−0.5	−1	−1	−0.8
4	−0.5	−1.5	−0.5	−1
5	−2	−1.5	−2.5	−2
6	−2	−1	−1.5	−1.5
7	1	−0.5	0.5	0.3
8	3	1	0.5	1.5
9	3	0.5	0.5	1.3
10	2	0.5	0.5	1

Total				1.15

**Table 3:** Mean and standard deviation of Broadrick Occlusal curve of subjects and control groups.

Group	Mean (mm)	Standard deviation	Range (mm)
Subjects	1.15	0.53	−2.5 to 3
Controls	0.88	0.04	0.5 to 1.5
